# The Patterns of Impacted Third Molars and Their Associated Pathologies: A Retrospective Observational Study of 704 Patients

**DOI:** 10.3390/jcm13020330

**Published:** 2024-01-06

**Authors:** Salha Othman Al-Madani, Mohamed Jaber, Prathibha Prasad, Manal Jamil Mohammad Al Maslamani

**Affiliations:** 1 Al-Fujairah Dental Center, Fujairah P.O. Box 2299, United Arab Emirates; salhaothman@yahoo.com; 2 Clinical Dental Sciences Department, College of Dentistry, Ajman University, Ajman 346, United Arab Emirates; mohamed.jaber@ajman.ac.ae (M.J.); m.almaslamani@ajman.ac.ae (M.J.M.A.M.); 3 Center of Medical and Bio-Allied Health Sciences Research, Ajman University, Ajman 346, United Arab Emirates; 4 Basic Dental Sciences Department, College of Dentistry, Ajman University, Ajman 346, United Arab Emirates

**Keywords:** extraction, impacted teeth, oral surgery, oral diagnosis, oral pathology

## Abstract

**Background:** The study aims to investigate parameters in patients attending Fujairah Dental Center, including assessing the prevalence of impacted teeth, determining the frequency of associated pathological conditions, and evaluating the patterns and angulations of impacted third molars. **Methods:** It is a retrospective descriptive study of the panoramic radiographs of patients who attended Fujairah Dental Center for dental care between January 2011 and December 2017. The minimum age for inclusion was 17 years. Clinical records were used to obtain the demographic details of patients, such as age, gender, nationality, medical history, and smoking history. Seven hundred and four panoramic radiographs and clinical records of patients were analyzed. The age range was between 17 and 112 years old, with a mean age of 34 years (S.D 13.5). **Results:** Of the 704 panoramic radiographs evaluated, 236 (33.6%) X-rays showed teeth impaction with a total of 562 impacted teeth in the upper and lower jaws. Five hundred and twenty-five (93.4%) were impacted third molars, and 37 (6.5%) teeth were other kinds of impacted teeth. Females showed a higher frequency of impacted teeth (62.6%) compared to males (37.4%). The highest prevalence of impacted teeth was found in the 17–25 year age group (61%), and the prevalence declined with advancing age. Impacted third molars were more likely to occur in the mandible (57.3%) than in the maxilla (42.7%). Most of the impacted third molars were in the mesioangular position, followed by the vertical position. The evaluation of the depth of impacted third molars demonstrated that level C impaction was the most frequently seen, followed by level B impaction. Impacted third-molar teeth often presented with two roots (60.7%), followed by a single root (31.3%). An evaluation of the relationship between lower third molars and the inferior dental canal (IDC) revealed that the most frequently observed relation was interrupted (61.5%), followed by a distant relation to the ID canal, and 13% were superimposed. The most common morphological pattern of roots of the impacted third molars was either straight-type or curved and dilacerated roots (5.7%). Additionally, the most noticed pathological conditions associated with impacted teeth were carious second molars adjacent to impacted third molars (42%), which was more likely to be seen in the 17–25-year age group, with females having a higher prevalence than males.

## 1. Introduction

The most common minor oral surgical procedure performed by oral and maxillofacial surgeons is third-molar surgery [1]. Peterson defined impacted teeth as those teeth that fail to erupt into their normal position in the jaws on expected eruption time for many reasons [2,3]. Alternatively, impacted teeth have been defined by Farman as those that are prevented from erupting into their proper positions by a physical barrier within their path [2]. Impaction is a cessation of the eruption of a tooth caused by a clinically or radiographically detectable physical barrier in the eruption path or by an ectopic position of the tooth [4].

The eruption time of third molars for females is 17.39 ± 2.273 years, and for males, it is 16 ± 2.123 years. The complete eruption of third molars is earlier in males with 20.33 ± 2.566 years [5]. Factors such as a lack of space, disturbances in skeletal growth, increased tooth size, and delayed maturation of the third molars result in their impaction [6]. The absence of symptoms associated with impacted third molars does not equate to the absence of pathology, as pericoronitis and dysodontiasis may still be present without major symptoms [7]. Furthermore, the presence or absence of pathologies of the third molar particularly influences the occurrence of alveolar osteitis after tooth extraction [8,9].

The management of impacted third molars remains a controversial issue. Many countries have developed guidelines that dictate how third-molar teeth should be managed [3]. To date, there is no available data on the prevalence of impacted teeth, patterns of third molar impaction, and associated pathologies among the UAE (United Arab Emirates) population [10].

The aim of the study is to investigate the following parameters in patients who attended Fujairah Dental Center: to assess the prevalence of impacted teeth among patients attending Fujairah Dental Center, to determine the frequency of pathological conditions that are associated with impacted teeth, and to evaluate the different patterns and angulations of impacted third molars.

## 2. Materials and Methods

The study proposal was reviewed and approved by the Ethical Committee of Hamdan Bin Mohammed Dental College/Mohammed Bin Rashid University of Medicine and Health Sciences (Reference number: EC0415-007) and by the Ministry of Health and Prevention Research Ethics Committee (Reference Number: MOHP/DXB/SUBC/NO-19/2016). All subjects gave their informed consent for inclusion before they participated in the study. A written informed consent for publication must be obtained from participating patients.

**Null** **Hypothesis.** *There is no significant variation in the prevalence of impacted teeth among patients attending Fujairah Dental Center concerning different age groups and genders, and this prevalence is not associated with an increased frequency of pathological conditions.*

### 2.1. Study Design, and Location

A retrospective study evaluating the panoramic radiographs of patients attending Al-Fujairah Dental Center for any dental care was completed. Patients’ clinical records and panoramic radiographs were retrieved for assessment. The study was conducted at the Al Fujairah Dental Center in AL Fujairah—United Arab Emirates.

Inclusion criteria:

- Patients’ files with complete demographic details of patients;
- Panoramic radiographs of diagnostic quality;
- UAE nationals and non-nationals;
- Males and females;
- Patients aged 17 years old and above.

Exclusion criteria:

- Lack of proper panoramic X-rays and proper documentation of demographical details of patients;
- Patients below 17 years of age.

Sample size calculation:

In the current study, all required data were used to determine the minimum sample size to obtain reliable statistical information to draw inferences about the whole population. Sample size (n) was calculated via the online OpenEpi link using the Kish formula for sample size estimation, choosing a 95% significance level, a 5% margin of error, and a 20,000 population size. The representative minimal sample size was 377.

#### 2.1.1. Data Collection

Panoramic radiographs and clinical records of patients who attended Al Fujairah Dental Center for any dental care between January 2011 and December 2015 were retrieved and evaluated. All demographic information was extracted from the dental records, such as age, gender, nationality, and smoking history. All panoramic radiographs were taken with a Myray Hyperion X9. One researcher examined digital panoramic radiographs from computer archives. The data were collected using a standardized form, and the data were entered into a Microsoft Office 2007 Excel sheet.

The data collection sheet used was divided into two sections:

A. Demographical details of patients.

Which included: year of birth, gender, nationality, medical history, date of radiographs taken, and smoking history.

B. Radiographical evaluation

The panoramic radiographs were reviewed for:

- Presence and/or absence of impacted teeth;
- Kinds of impacted teeth;
- Number of impacted teeth in both arches;
- Angulation patterns of impacted third molars;
- Depth of impacted third molars;
- Kind of pathology present in or around the impacted teeth;
- Variation in the number of roots of impacted third molars;
- Variation in morphological patterns of the roots of the impacted third molars;
- The relation of inferior dental canals to impacted mandibular third molars.

The angulations of impacted third molars were evaluated based on Winter’s classification, with reference to the angle formed between the intersected longitudinal axes of the second and third molars [6]. The types of angular inclination used are as follows:

- Mesioangular;
- Distoangular;
- Vertical;
- Horizontal;
- Inverted and others.

The depth of the third molars was classified according to the Pell–Gregory classification [6].

Level A: the highest part of the third molar was on the same level or above the occlusal plane of adjacent second molar.
Level B: the highest part of the third molar was below the occlusal plane but above the cervical line of second molar.
Level C: the highest part of the third molar was beneath the cervical line of the second molar.

#### 2.1.2. Ectopic Position

The relation of the root of impacted third molars to the interdental canal was also examined and categorized into:

- Distant;
- Superimposed;
- Interrupted.

The number and morphological patterns of the roots of impacted third molars were also examined in this study.

The number of roots was assessed and classified into:

- One root;
- Two roots;
- Three roots;
- Roots cannot be assessed.

The morphological patterns of the roots of impacted third molars were evaluated and categorized into:

- Curved roots;
- Dilacerated roots;
- Straight roots;
- Fused roots;
- Roots cannot be assessed.

#### 2.1.3. Statistical Considerations and Data Analysis

Data was entered into a computer using SPSS (Statistical Package for Social Science) for Windows version 24.0 (SPSS Inc., Chicago, IL, USA). The results were cross-tabulated to examine the independence between variables. Statistical analysis was performed using the χ^2^-test and Fisher’s exact test as appropriate. Where two or more continuous independent variables were examined, a *t*-test and analysis of variance were used as was adequate. A *p*-value of less than 0.05 was considered significant in all statistical analyses.

## 3. Results

Seven hundred and four panoramic radiographs and clinical records of patients were reviewed. Clinical records were used to obtain the demographic details of patients, such as age, gender, nationality, medical history, and smoking history. The patients in this study were between 17 and 112 years old, with a mean age of 34.18 years (SD 13.5). Of the 704 panoramic radiographs evaluated, 468 (66.4%) patients showed no impacted teeth, and 236 (33.6%) patients possessed an impacted tooth.

A total of 562 impacted teeth were identified, of which 527 (93.7%) were impacted third molars, and 35 (6.2%) were other kinds of impacted teeth. 

### 3.1. The Demographical Details of Patients and the Presence of Impacted Teeth 

#### 3.1.1. Nationality

Our study found that the prevalence of impacted teeth among UAE-national patients was 207 (34.7%), while 29 (26.8%) were non-UAE-national patients (Table 1). There was no statistical correlation between the prevalence of impacted teeth and the nationality of the patients (*p*-value = 0.11). 

#### 3.1.2. Smoking History 

There was no association between the prevalence of impacted teeth and the smoking history of patients (as illustrated in Table 2), *p*-value = 0.33.

#### 3.1.3. Gender

Our study showed that 98 males (35.6%) had impacted teeth compared with 177 (64.4%) without impacted teeth. Among the 429 females examined, only 138 (32.2%) had impacted teeth, and 67.8% had no impacted teeth. There was no statistically significant difference between the prevalence of impacted teeth and gender (*p*-value = 0.3415) (Table 3).

### 3.2. Impacted Teeth and Age Groups 

**Figure 1:**
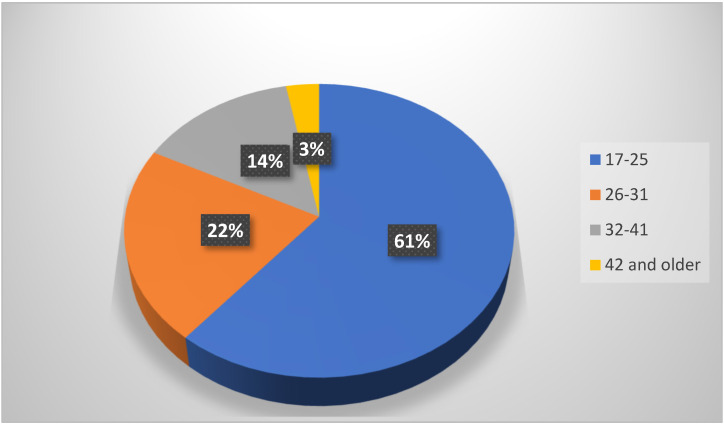
Age groups and prevalence of impacted teeth.

The highest prevalence of impacted teeth occurred in the 17–25 year age group, with 144 patients who had 404 impacted teeth, followed by 26–31 year olds with 51 patients, who had 102 impacted teeth, and this declined gradually with advancing age to 14.4% at age 26–31 years and 3% in the age group 42 years and older (Figure 1).

### 3.3. The Distributions of Patients with Impacted Teeth and the Correlation with Gender 

**Figure 2:**
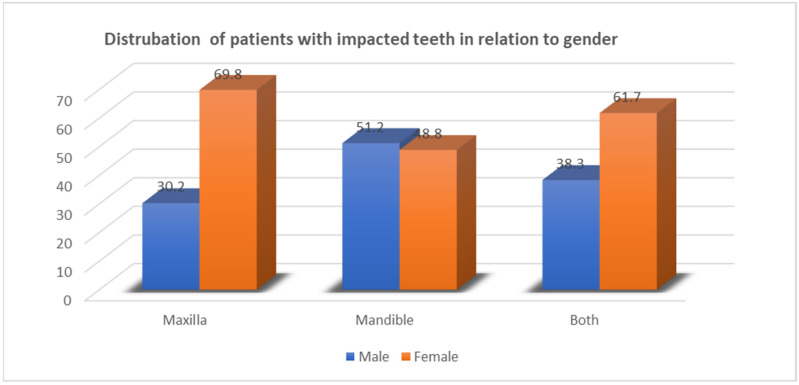
Distributions of patients with impacted teeth in relation to gender. Chi-Square (χ^2^) = 6.0013 (*p*-value = 0.04).

There was a statistical association between the prevalence of impacted teeth and gender in relation to the jaws (*p*-value = 0.04). Thirty females (69.8%) had only impacted teeth in the maxillary arch, in comparison to thirteen males (30.2%). 51.2% males and 48.8% females had only mandibular impacted teeth. Furthermore, 61.7% of females and 38.3% of males showed impaction in both the jaws (Figure 2).

### 3.4. Impacted Third-Molar Teeth in Both Jaws 

Of 527 impacted third-molar teeth, there were 225 (42.7%) maxillary third molars and 302 (57.4%) mandibular third molars (*p* < 0.001).

### 3.5. Impacted Third Molars and Gender 

Of the 527 impacted third molar teeth, there were 202 (38.3%) impacted teeth among males and 325 (61.6%) among females. The male (M) to female (F) ratio of impacted third molars was 1:1.6, with a statistically significant difference between the two genders (*p* < 0.001). 

### 3.6. The Angulation of Impacted Third Molars

The most commonly seen pattern of the angulations of impacted third molars was mesioangular (182 (34.5%)), followed by vertical [145 (27.5%)], 143 (27%) distoangular and 47 (9%) horizontal.

#### 3.6.1. The Angulation of Impacted Third Molars in Both Jaws (Table 4)

In the maxillary arch, distoangular impaction was more prevalent (91 teeth, 40.4%), followed by vertical position (65 teeth, 28.9%). However, in the lower jaw, most of the impacted third molars (125, 41.4%) were in mesial inclination, followed by the vertical position (80, 26.5%). There was a statistical association between the angulation of impacted third molars and the jaws involved (*p* < 0.001).

**Table 4:** Distribution of different angulations of impacted third molars in both jaws.

Angulations	Maxilla	Mandible	Total
Mesioangular	57 (25.3%)	125 (41.4%)	182
Distoangular	91 (40.4%)	52 (17.2%)	143
Vertical	65 (28.9%)	80 (26.5%)	145
Horizontal	3 (1.33%)	44 (14.6%)	47
Ectopic/other	9 (4%)	1 (0.3%)	10
Total	225 (100%)	302 (100%)	527

Chi-Square (χ^2^) = 70 (*p* < 0.001).

#### 3.6.2. The Angulation of Impacted Third Molars and Gender (Table 5)

Our observation showed that mesioangularly impacted third molars were more common in females (124, 38.2%) than males (58, 28.7%). The second most common angular position seen in females was distoangular (96, 29.5%), followed by vertical (76, 23.4%). On the other hand, males showed that the most frequently seen angular position of impacted third molars was vertical (69, 34.2%), followed by mesioangular (58, 28.7%), and distoangular (47, 23.2%). There was a significant association between the angulation of impacted third molars and gender (*p* < 0.009).

**Table 5:** Distribution of different angulation of impacted third molars in relation to gender.

Angulations	Males	Females	Total
Mesioangular	58 (28.7%)	124 (38.2%)	182
Distoangular	47 (23.2%)	96 (29.5%)	143
Vertical	69 (34.2%)	76 (23.4%)	145
Horizontal	24 (11.9%)	23 (7.1%)	47
Ectopic/other	4 (2%)	6 (1.8%)	10
Total	202 (100%)	325 (100%)	527

Chi-Square (χ^2^) = 13.511 (*p* = 0.009).

### 3.7. The Depth of Impacted Third Molars

An assessment of the level of impaction of third molars using the Pell and Gregory classification demonstrated that most of the impacted third molars, 251 (48%), were positioned at level C, 174 (33%) at level B, and 85 (16%) at level A.

#### 3.7.1. The Depth of Impacted Third Molars in Both Jaws

Our present study showed that the level C impaction of third-molar teeth showed a higher tendency to occur in the maxilla (152, 67.6%) than in the mandible (99, 32.8%) (Table 6).

On the other hand, level B-impacted third molars were the most prominent in the mandible (116, 38.5%). There was a statistical correlation between the depth levels of impacted third molars and the jaw involved (*p* < 0.001).

#### 3.7.2. The Depth of Impacted Third Molars and Gender 

Type C-impacted third molars were the most frequently observed depth level in both genders.

Females showed 154 (47.4%) and males showed 98 (48.1%) impacted third molars. There was no significant association between the levels of impacted third molars and gender (*p* = 0.158) (Table 7).

### 3.8. The Roots of Impacted Third Molars and the Relationship with Inferior Dental Canal

An evaluation of the relationship between lower third-molar roots and inferior dental canals showed that the most frequently observed relation as seen in both genders was an interrupted relation in 186 teeth (61.5%), followed by a distant relation in 77 (25.5%) from the interdental canal, and 39 (13%) were superimposed.

#### The Relationship between Inferior Dental Canals and the Roots of Impacted Third Molars

Females showed a higher prevalence of interrupted relations (58.8%), followed by distant relations (26.2%), and the least common was the superimposed position (15%) (Table 8).

Among males, the most commonly noticed kind of relation between the roots of impacted third molars and the interdental canal was interrupted (65.9%), followed by distant (24.3%) and superimposed (9%).

There was no statistically significant difference between gender and the relation between the roots of wisdom teeth and an inferior dental canal, *p* value = 0.314.

### 3.9. The Number of Roots of Impacted Third Molars

The greater distribution of the number of roots of impacted third molars was two roots in 319 teeth (60.7%), followed by single roots in 165 teeth (31.3%), and the least was three roots (2%).

#### The Number of Roots of Impacted Third Molars in Both Jaws (Figure 3)

There was a statistically significant difference between the number of roots of impacted third molars and jaws (*p* < 0.001). The majority of upper third molars had single-rooted teeth (149 (66.2%)), followed by two roots, and only 1.8% were three-rooted teeth. On the other hand, the two-rooted third molars were most frequently seen in the mandible, with 269 teeth (89.1%), followed by single-rooted teeth (5.2%).

**Figure 3:**
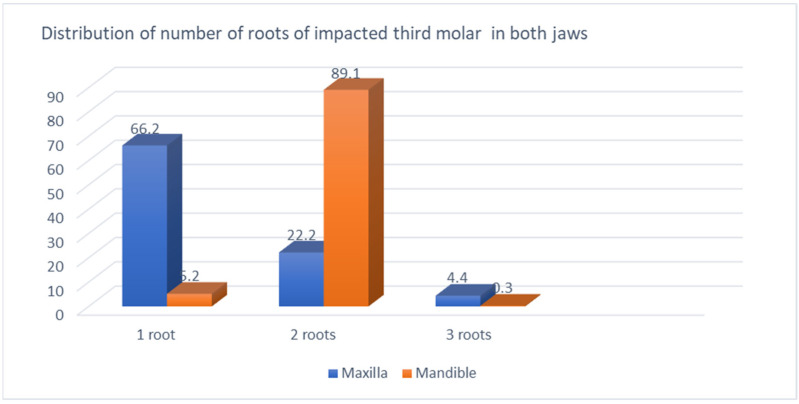
Distribution of number of roots of impacted third molar in both jaws. Chi-Square (χ^2^) = 259.20 (*p* < 0.0001).

### 3.10. The Morphological Pattern of Impacted Third-Molar Roots

The most common morphological pattern noticed in both jaws was straight roots (284 teeth, 53.9%) followed by curved roots (151 (28.7%)). The prevalence of dilacerated roots was 30 teeth (5.7%); all of them were seen in the mandible.

#### The Morphological Pattern of Impacted Third-Molar Roots in Both Jaws (Figure 4)

There was a statistical correlation between the jaws and the morphological pattern of the roots of impacted third molars (*p* < 0.001). The curved roots of impacted third molars (40.7%) were most frequently seen in the mandible, followed by straight roots (35.7%), and 10% were dilacerated roots. In the maxilla, the most prominent root morphology of impacted third molars was straight roots (78.2%), followed by curved roots (12.4%).

**Figure 4:**
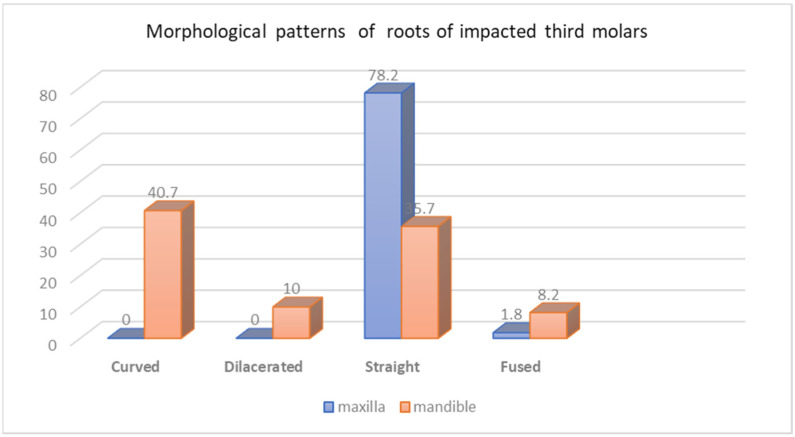
Morphological patterns of roots of impacted third molars. Chi-Square (χ^2^) = 112.43 (*p*-value = *p* < 0.0001).

### 3.11. Pathological Conditions Associated with Impacted Third Molars

The prevalence of pathological conditions that may be associated with the impacted teeth revealed that the number of patients with carious second molars adjacent to the impacted third molars was 99 (42%), and the patients without carious adjacent molars was 137 (58%). 

Females had a (25%) higher prevalence than males (40 (17%)), with a *p* value of 0.003.

#### 3.11.1. The Dental Caries of Second Molars Adjacent to Impacted Teeth in Males (Table 9)

There was statistical significance between the prevalence of carious second molars and males, (*p* = 0.0033). Males in the youngest age group showed the highest prevalence of carious second molars adjacent to impacted third molars (50%), followed by 32.5% in the second age group (26–31 years old), and the prevalence decreased with advancing age.

**Table 9:** Dental caries of second molars adjacent to impacted teeth among males.

	Males		
Age Group	Patients with Carious Second Molars	Patients without Carious Second Molars	Total
17–25 years	20 (50%)	34 (58.6%)	54
26–31	13 (32.5%)	8 (13.8%)	21
32–41	7 (17.5%)	11 (19%)	18
42 and above	0	5 (8.6%)	5
Total	40 (100%)	58 (100%)	98

Chi-Square (χ^2^) = 13.71 (*p*-value = 0.0033).

#### 3.11.2. The Dental Caries of Second Molars Adjacent to Impacted Teeth in Females (Table 10)

Among females, the majority of carious second molars (69.5%) were seen in the youngest age group (17–25 years), followed by (20.3%) and (8.5%) in the second and third age groups, respectively. 

**Table 10:** Dental caries of second molars adjacent to impacted teeth among females.

	Females		
Age Group	Patients with Carious Second Molars	Patients without Carious Second Molars	Total
17–25 years	41 (69.5%)	48 (60.8%)	89
26–31	12 (20.3%)	18 (22.7%)	30
32–41	5 (8.5%)	11 (14%)	16
42 and above	1 (1.7%)	2 (2.5%)	3
Total	59 (100%)	79 (100%)	138

Chi-Square (χ^2^) = 1.4661 (*p*-value = 0.6901).

### 3.12. Distribution of Patients with Impacted Teeth and Associated Pathological Conditions (Table 11)

Twenty-two patients (9.3%) had caries in the impacted third molars. Additionally, there were 22 patients (9.3%) with pericoronal radiolucency around the crowns of impacted teeth, while 9 patients (3.8%) showed the presence of periapical radiolucency around the roots of impacted teeth. Furthermore, the results showed that 12 patients (5%) were associated with the root resorption of adjacent second molars, and only 2 patients (1%) had root resorptions of their impacted third molars.

**Table 11:** Distribution of patients with impacted teeth and associated pathological conditions.

Kind of Pathosis	No. of Patients with Pathosis	Percent %	No. of Patients without Pathosis	Percent %	Total	*p* Value
Dental caries of adjacent second-molar teeth	99	42	137	58	236	0.005
Dental caries of impacted third molars	22	9.3	214	90.7	236	*p* < 0.001
Root resorption of second molars	12	5	224	95	236	*p* < 0.001
Root resorption of impacted third molars	2	0.8	234	99.2	236	*p* < 0.001
Periapical radiolucency	9	3.8	227	96.2	236	*p* < 0.001
Pericoronal radiolucency	22	9.3	214	90.7	236	*p* < 0.001

#### The Association between Dental Pathologies and Specific Dental Conditions (Table 11)

The dental caries of adjacent second molars is significantly associated with the presence of the pathology, with a *p*-value of 0.005. In total, 42% of patients with pathosis had dental caries of the adjacent second molars, compared to the 58% without pathosis. Dental caries in impacted third molars is strongly associated with the presence of the pathology, with a *p*-value less than 0.001. Only 9.3% of patients with pathosis did not have dental caries of the impacted third molars, while 90.7% of patients without pathosis did not have dental caries of impacted third molars.

The root resorption of second molars is strongly associated with pathology, with a *p*-value of less than 0.001. Only 5% of patients with pathosis did not have root resorption of the second molars, while 95% of patients without pathosis did not have root resorption of the second molars.

The root resorption of impacted third molars is strongly associated with the pathology, with a *p*-value of less than 0.001. Only 0.8% of patients with pathosis did not have root resorption of the impacted third molars, while 99.2% of patients without pathosis did not have root resorption of the impacted third molars.

Periapical radiolucency and pericoronal radiolucency are strongly associated with the presence of the pathology, with *p*-values of less than 0.001. In total, 96.2% and 90.7% of patients without pathosis did not have periapical radiolucency and pericoronal radiolucency, respectively.

## 4. Discussion

Teeth that fail to erupt into their normal position in the jaws on the expected eruption time due to many reasons are considered impacted [11]. These teeth may stay intact throughout the life of the person with no signs or symptoms. However, at any time, pathological conditions may develop with the association of the impacted teeth, which indicates the necessity for the removal of the impacted teeth [12]. However, the removal of impacted teeth may carry some risks and complications that affect the quality of life of patients in the short term or even for the long term [13]. Investigating the prevalence of impacted teeth, including third molars, angulation and depth, variation in morphology and number of roots, the relationship to the interdental canal, and other clinically significant findings will help in reducing the complications that may occur during teeth extraction [14].

Our study found that the prevalence of impacted teeth was 33.6 percent, as reviewed from panoramic radiographs of patients attending Fujairah Dental Center. These results were similar to a study conducted by El-Khateeb et al., in which they reported that 34.5 percent of patients had impacted teeth and 65.5 percent had no impaction [15]. However, our results were slightly less than those of Hassan, who reported that the prevalence was 40.5% in the western region of Saudi Arabia [16]. A pilot study on the Iraqi population also showed a 46.7% prevalence [17]. But, Byahatti and Ingafou reported a slightly lower (27.9%) prevalence of impacted teeth among Libyan students [18]. Also, in the south region of Saudi Arabia, the prevalence was low (18.76%), as reported by Syed et al. [19]. Additionally, Al-Anqudi et al. conducted a study in Oman, and they found that the prevalence of impacted teeth was 54.3% [20].

Internationally, the prevalence of impaction has been investigated by many authors: by Pillai et al. for the Indian population (56.52%), and by Kalliopi et al. for the Greek population (15.14%) [21,22].

The most common teeth that fail to erupt, as seen in this study, were the third molars (93.7%), which agreed with Jain. Where the prevalence was 52.3%, agreeing with Salam, it represented 22% of all dental impactions [23,24]. Furthermore, other studies reported a high frequency of impacted third molars, as illustrated by Šečić et al. for the Bosnia and Herzegovina population (51.7%), Ishwarkumar for the South African Indian population (81%), and in a study by Raj Kumar, where the prevalence was 79.7% [25,26,27]. Compared to other populations, UAE residents seem to demonstrate a higher percentage of impacted third molars. This could be due to the fact that most residents are expats and belong to different nationalities.

The present study showed a significant association between the prevalence of impacted third molars and gender. Females showed a higher frequency of impacted third molars (61.6%) than males (38.3%). This finding coincided with several studies in which females had a higher prevalence of impacted third molars than males [28]. Al-Anqudi et al. described the prevalence as 60% among females and 40% among males [20]. On the other hand, Syed et al. and Gupta et al. found no difference in the prevalence of impaction in both the genders [19,29]. But, Jung and Cho found that impaction was more frequent in males [30]. Some authors have attributed this difference to the fact that the jaws of females stop growing at the time when mandibular third molars are just beginning to erupt, and consequently, there is frequently insufficient space for them. In males, the growth of the jaws continues beyond the time of the eruption of the third molar; therefore, more space is provided with fewer impactions [31].

The current study showed that 57.3% of the sample were impacted mandibular third molars, which is considered to be higher than that of impacted maxillary third molars (42.7%). According to Hatem et al., the prevalence of impacted lower-third molars was 56% and that of impacted upper-third molars was 44% [32]. A number of studies found that the prevalence of impacted third molars was higher in the mandible [28]. There is still debate about whether the impaction of the mandibular third molar or maxillary third molar is more commonly seen [33]. It is generally agreed that the mandibular third molar becomes impacted more than any other tooth; from 17% to 25% of the general population will have one or more impacted third molars [34]. The difference may be due to sampling variations, racial characteristics, and different methods of study.

In this study, the majority of patients (61%) who had impacted teeth were in the young age group (17–25 years old), and prevalence decreased with advancing age. This was consistent with Syed [19]. 

The mesial inclination of impacted third molars was the most commonly observed angular pattern in this study (34.5%), which was consistent with Hatem et al., in which the prevalence was 34.6% [32]. Other studies were also consistent with our findings [20,34]. Other countries reported higher figures, like 50.71% in Saudi Arabia, 70.3% in India, and 50% in Malaysia [19,21]. 

On the other hand, our findings were different from El-Khateeb et al., Byahatti and Ingafou, who reported that in the lower arch, the most frequent angular pattern detected was the vertical position [15,18]. Jung and Cho found that the horizontally impacted third molars were mostly seen in the mandible [30]. 

In the current study, the most common angular pattern of impacted third molars in the maxilla was distal inclination (40.4%). El khateeb et al. and Hashemipour et al. reported that vertically positioned third molars were most commonly seen in the maxilla [15,34]. Our population also showed a different angulation than the Libyan population [18]. Residents from other emirates in the UAE should be studied to correlate the data.

The angulation of impacted third molars showed a significant association with gender (*p* value = 0.009) in such a way that females showed a predominance in mesioangular impaction (38.2%), while males showed a predominance in vertical impaction (34.2%). This was in contrast to Hatem et al., who found that the most predominant angulation seen in females was distoangular, while in males, it was mesioangular [32].

Our observation showed that the most prominent depth of impacted third molars was level C impaction (48%), followed by level B impaction (33%). These findings are in contrast to other studies conducted by Hassan, who found that among the Saudi population, level B impaction was the most common and the least frequent was level C [16]. Likewise, Hatem et al. found that level B (44.7%) was most common and the least common was level C impaction (29%) among the Libyan population [32]. However, other studies revealed that depth A was the most common and the least common was level C impaction [20]. 

The jaw-wise distribution and depth of impacted third molars was investigated in this study. It showed that Level B (38.5%) was the most frequently observed in the mandible. On the other hand, Passi et al. showed that the level B impaction of third molars was mostly seen in the mandible (64.2%) [35]. It was found in our study that 67.6% of impacted upper-third molars were positioned at level C, which displayed the highest proportion compared with other depth levels seen in the maxilla. This is in agreement with other studies [15,30,32].

Similarly, Shaari et al. found that in the maxilla, the highest frequency seen was level C [14]. The disparity in the depth position and angulation of impacted third molars could be attributed to the racial variation, sample size, and methodology of their research.

In the current study, the relationship between the roots of impacted third molars and inferior dental canals revealed that the most frequently seen relation type was interrupted (61.5%), followed by a distant relationship between roots and the canal (25.4%), and 39 (12.9%) were superimposed. Ishak et al. found that interruption of the radiopaque line of the inferior dental canal (55.6%) was higher than superimposition (35.3%) [36]. Likewise, Deshpande et al. showed that the mean distance of the impacted third molar from the inferior dental canal was 0.50mm (61.8%) [37].

In the current study, two-rooted (89.1%) impacted third molars were most commonly seen in the mandible, followed by the single-rooted teeth (5.2%). These results were in accordance with Kuzekanani et al., who showed that the majority of lower-third molars had two roots (73%), 21% had one root, and only 5.5% had three roots [38]. Park et al. conducted a study among the Korean population and indicated that most of the mandibular third molars either had two roots (56.5%) or one root (37.9%) [39]. Our study showed that single-rooted impacted third molars were mostly occurred in the maxilla (66.2%). This was in agreement with Jung and Cho, who found that 46% of maxillary third molars were single-rooted [30]. But, this is in contrast to Ahmad et al., who reported that among the Jordanian population, the majority of impacted maxillary third molars (74.2%) had three roots and 13.5% were single-rooted [40]. 

The morphological pattern of the roots of third molars in this study showed that the most prominent types were straight roots (53.9%), followed by curved roots (28.7%), and (5.7%) dilacerated roots. This is in contrast to Bokindo et al., who reported among the Kenyan population that most of the roots were dilacerated (44%), and the 1:1 ratio was straight and dilacerated [41]. In the current study, there was no prevalence of dilacerated roots in the maxilla, and only 6% of roots were dilacerated in the lower jaw. The clear disparity among the number and pattern of roots of the third molar could also be attributed to the racial variation, sample size, and methodology of their research.

In the present study, the pathological lesions that may occur in association with impacted teeth were investigated. Our results demonstrated that the majority of pathological conditions occurred in the younger age group years (17–25). These findings were in agreement with Pursafar et al., who reported that the incidence of pathologies associated with impacted teeth was mainly seen in the 21–30 year old-age group [42].

The current study showed that pericoronal radiolucency around the impacted third molars was noted in 9.3% of the cases, while pericoronal radiolucency associated with impacted third molars was seen in 9.3% of the cases, which was similar to that reported by Tassoker et al. [43].

Furthermore, our findings showed that 5% of second molars adjacent to impacted third molars had root resorption. Pursafar et al. reported that 1.4% of second molars had root resorption [42]. The prevalence of root resorption in impacted third molars was low; only one percent was found in the current study. Sejfija et al. found that the resorption of the roots of the third molars was 23.7% [44].

Our observation in this study showed that the prevalence of dental caries of the second molars adjacent to the impacted third molars was 42%. This was similar to findings by Syed et al. (39%) and 38% by Toedtling et al. [16,45]. The proportion was 25.5% among the Brazilian population [46]. These results were greater than the findings of Pursafar et al., (1.6%) [42]. 

Our study showed that 8.3% had dental caries of the impacted third molars, which was close to Oyebunmi et al.’s outcomes [47]. Al-Anqudi et al. found that four percent of impacted lower-third molars had dental caries [20]. On the other hand, Pentapati et al. reported a higher prevalence of dental caries in impacted teeth [48]. 

The majority of carious second molars appeared in the youngest age group (17–25 years), followed by 26–31 years (26% and 10.6%, respectively), and the prevalence decreased with increasing age. Our observation was in agreement with previous studies, which found that the majority of carious second molars were in the younger age groups of 16–30 years [49]. In contrast to other studies that found a higher prevalence of second molar caries with advancing age, this was attributed to an increased chance for plaque accumulation and bacteria to cause caries at the distal aspect of the second molars adjacent to the impacted third molars [50]. The assessment of carious second molars adjacent to impacted third molars between different age groups and genders showed that males (50%) and females (69.5%) in the youngest age group had the highest prevalence of carious second molars, and it decreased in the older age group. Additionally, males showed a statistically significant association between age and the prevalence of carious second molars adjacent to impacted third molars (*p* = 0.033) [51]. Syed et al. reported that males (83%) had a greater tendency to have distal caries of the second molars than females (17%), which was attributed to the frequency of oral hygiene measures and the possibility related to the type of food consumed and regular visits to the dentist [49].

### Limitations of the Study

This is a retrospective study based on patients’ records and panoramic radiographs. Thus, it may be subjected to many limitations, like missing radiographs from the patients’ files, the incomplete documentation of the patients’ demographic details, and radiographs of poor quality. Furthermore, panoramic radiographs are widely used in dentistry; however, many problems may arise from using such types of radiographs, like the magnification of images, the superimposition of adjacent structures, and the fact that they are two-dimensional images.

## 5. Conclusions

The findings from this study will contribute to improved diagnosis and treatment planning for patients with impacted third molars. This is the first study performed to provide a glimpse of the current status of the impacted teeth in a UAE population; such data can be used to support or refute the routine removal or retention of the impacted teeth. Furthermore, this study may help in developing national guidelines and protocols for the management of impacted teeth and associated pathologies. Future studies are needed to evaluate the prevalence of impaction, different patterns of third-molar impaction, and the pathological conditions that may be associated with impacted teeth in other regions of the UAE.

## Figures and Tables

**Table 1:** Nationality and prevalence of teeth impaction.

	Nationality		
Impaction Status	UAE Nationals %	Non-UAE Nationals %	Total %
Impacted	207 (34.7%)	29 (26.8%)	236
Non-Impacted	389 (65.2%)	79 (73.2%)	468
Total	596	108	704

Chi-Square (χ^2^) = 2.5474 (*p*-value = 0.1105).

**Table 2:** Smoking history and prevalence of teeth impaction.

	Smoking History		
Impaction Status	Non-Smoking %	Smoking %	Total %
Impacted	18 (28.1%)	218 (34%)	236
Non-Impacted	46 (71.8%)	422 (66%)	468
Total	64	640	704

Chi-Square (χ^2^) = 0.9204 (*p*-value = 0.3376).

**Table 3:** Gender and prevalence of teeth impaction.

	Gender		
Impaction Status	Males	Female	Total
Impacted	98 (35.6%)	138 (32.2%)	236
Not impacted	177 (64.4%)	291 (67.8%)	468
Total	275	429	704

Chi-Square (χ^2^) = 0.9047 (*p*-value = 0.3415).

**Table 6:** Distribution of different depth levels of impacted third molars in both jaws.

Depth	Maxilla	Mandible	Total
Type A	2 (0.9%)	83 (27.5%)	85
Type B	58 (25.8%)	116 (38.5%)	174
Type C	152 (67.6%)	99 (32.8%)	251
Ectopic/other	13 (5.7%)	3 (1%)	16
Total	225 (100%)	302 (100%)	527

Chi-Square (χ^2^) = 105.177 (*p* < 0.001).

**Table 7:** Distribution of different depth levels of impacted third molars in relation to gender.

Depth	Males	Females	Total
Type A	39 (19.3%)	46 (14.2%)	85
Type B	62 (30.7%)	112 (34.4%)	174
Type C	98 (48.1%)	154 (47.4%)	252
Ectopic/other	3 (1.5%)	13 (4%)	16
Total	202 (100%)	325 (100%)	527

Chi-Square (χ^2^) = 5.1953. (*p* = 0.158).

**Table 8:** Relationship between inferior dental canal and roots of impacted third molars.

	Males		Females		Total
Types	No	Percent %	No	Percent %	
Distant	30	24.3	47	26.2	77
Superimposed	12	9.8	27	15	39
Interrupted	81	65.9	105	58.8	186
Total	123	100	179	100	302

Chi-Square (χ^2^) = 2.3147 (*p*-value = 0.3143).

## Data Availability

The datasets used and analyzed during the current study are available from the corresponding author on reasonable request.

## References

[1] Sifuentes-Cervantes J.S., Carrillo-Morales F., Castro-Núñez J., Cunningham L.L., Van Sickels J.E. (2021). Third molar surgery: Past, present, and the future. Oral Surg. Oral Med. Oral Pathol. Oral Radiol..

[2] Juodzbalys G., Daugela P. (2013). Mandibular third molar impaction: Review of literature and a proposal of a classification. J. Oral Maxillofac. Res..

[3] Santosh P. (2015). Impacted Mandibular Third Molars: Review of Literature and a Proposal of a Combined Clinical and Radiological Classification. Ann. Med. Health Sci. Res..

[4] Carter K., Worthington S. (2015). Predictors of Third Molar Impaction. J. Dent. Res..

[5] Putul M., Konwar R., Dutta M., Basumatary B., Rajbongshi M.C., Thakuria K.D., Sarma B. (2021). Assessment of Age at the Stages of the Eruption of Third Molar Teeth among the People of North-Eastern India. BioMed Res. Int..

[6] Yilmaz S., Adisen M.Z., Misirlioglu M., Yorubulut S. (2016). Assessment of Third Molar Impaction Pattern and Associated Clinical Symptoms in a Central Anatolian Turkish Population. Med. Princ. Pract..

[7] Chisci D., Parrini S., Baldini N., Chisci G. (2023). Patterns of Third-Molar-Pericoronitis-Related Pain: A Morphometrical Observational Retrospective Study. Healthcare.

[8] Tolstunov L. (2012). Influence of immediate post-extraction socket irrigation on developmentof alveolar osteitis after mandibular third molar removal: A prospective split-mouth study, preliminary report. Br. Dent. J..

[9] Tolstunov L. (2013). Third molar uncertainty. J. Oral Maxillofac. Surg..

[10] Alsaegh M.A., Abushweme D.A., Ahmed K.O., Ahmed S.O. (2022). The pattern of mandibular third molar impaction and its relationship with the development of distal caries in adjacent second molars among Emiratis: A retrospective study. BMC Oral Health.

[11] Jaroń A., Trybek G. (2021). The Pattern of Mandibular Third Molar Impaction and Assessment of Surgery Difficulty: A Retrospective Study of Radiographs in East Baltic Population. Int. J. Environ. Res. Public Health.

[12] Qassadi T.M., Shafei A.A., Alhazmi A.A., Odabi N.I. (2020). Prevalence and pattern of third molar impaction among the Saudi population in Jazan region, Saudi Arabia. Saudi J. Oral Dent. Res..

[13] Chen Y.W., Chi L.Y., Lee O.K.S. (2021). Revisit incidence of complications after impacted mandibular third molar extraction: A nationwide population-based cohort study. PLoS ONE.

[14] Shaari R.B., Awang Nawi M.A., Khaleel A.K., AlRifai A.S. (2023). Prevalence and pattern of third molars impaction: A retrospective radiographic study. J. Adv. Pharm. Technol. Res..

[15] El-Khateeb S.M., Arnout E.A., Hifnawy T. (2015). Radiographic assessment of impacted teeth and associated pathosis prevalence. Pattern of occurrence at different ages in Saudi male in Western Saudi Arabia. Saudi Med. J..

[16] Hassan A.H. (2010). Pattern of third molar impaction in a Saudi population. Clin. Cosmet. Investig. Dent..

[17] Hasan L.S., Ahmad F.T., Abdullah E.H. (2016). Impacted Wisdom Teeth, Prevalence, Pattern of Impaction, Complications and Indication for Extraction: A Pilot Clinic Study in Iraqi Population. Tikrit J. Dent. Sci..

[18] Byahatti S., Ingafou M.S.H. (2012). Prevalence of eruption status of third molars in Libyan students. Dent. Res. J..

[19] Syed K.B., Zaheer K.B., Ibrahim M., Bagi M.A., Assiri M.A. (2013). Prevalence of Impacted Molar Teeth among Saudi Population in Asir Region, Saudi Arabia—A Retrospective Study of 3 Years. J. Int. Oral Health.

[20] Al-Anqudi S.M., Al-Sudairy S., Al-Hosni A., Al-Maniri A. (2014). Prevalence and Pattern of Third Molar Impaction: A retrospective study of radiographs in Oman. Sultan Qaboos Univ. Med. J..

[21] Kumar Pillai A., Thomas S., Paul G., Singh S.K., Moghe S. (2014). Incidence of impacted third molars: A radiographic study in People’s Hospital, Bhopal, India. J. Oral Biol. Craniofac. Res..

[22] Siotou K., Kouskouki M.P., Christopoulou I., Tsolakis A.I., Tsolakis I.A. (2022). Frequency and Local Etiological Factors of Impaction of Permanent Teeth among 1400 Patients in a Greek Population. Dent. J..

[23] Jain S., Debbarma S., Prasad S.V. (2019). Prevalence of impacted third molars among orthodontic patients in different malocclusions. Indian J. Dent. Res..

[24] Salam S., Bary A., Sayed A. (2023). Prevalence of Impacted Teeth and Pattern of Third Molar Impaction among Kerala Population a Cross Sectional Study. J. Pharm. Bioallied. Sci..

[25] Šečić S., Prohić S., Komšić S., Vuković A. (2013). Incidence of impacted mandibular third molars in population of Bosnia and Herzegovina: A retrospective radiographic study. JHSCI.

[26] Ishwarkumar S., Pillay P., Haffajee M.R., Satyapal K.S. (2019). Prevalence of Impacted Third Molars in the South African Indian Population of the eThekwini Metropolitan Region. SADJ.

[27] Kumar V.R., Yadav P., Kahsu E., Girkar F., Chakraborty R. (2017). Prevalence and Pattern of Mandibular Third Molar Impaction in Eritrean Population: A Retrospective Study. J. Contemp. Dent. Pract..

[28] Sivaramakrishnan S.M., Ramani P. (2015). Study on the Prevalence of Eruption Status of Third Molars in South Indian Population. Biol. Med..

[29] Gupta S., Bhowate R.R., Nigam N., Saxena S. (2011). Evaluation of impacted mandibular third molars by panoramic radiography. ISRN Dent..

[30] Jung Y.H., Cho B.H. (2013). Prevalence of missing and impacted third molars in adults aged 25 years and above. Imaging Sci. Dent..

[31] Singh P., Nath P., Bindra S., Rao S.S., Reddy K.V.R. (2018). The predictivity of mandibular third molar position as a risk indicator for pericoronitis: A prospective study. Natl. J. Maxillofac. Surg..

[32] (2016). Pattern of third molar impaction in Libyan population: A retrospective radiographic study. Saudi J. Dent. Res..

[33] Genç B.G.Ç., Orhan K., Hıncal E. (2022). Maxillary and mandibular third molars impaction with associated pathologies in a North Cyprus population: A retrospective study. Appl. Sci..

[34] Hashemipour M.A., Tahmasbi-Arashlow M., Fahimi-Hanzaei F. (2013). Incidence of impacted mandibular and maxillary third molars: A radiographic study in a Southeast Iran population. Med. Oral Patol. Oral Cir. Bucal..

[35] Dubey M., Passi D., Singh G., Dutta S., Srivastava D., Chandra L., Mishra S., Srivastava A. (2019). Study of pattern and prevalence of mandibular impacted third molar among Delhi-National Capital Region population with newer proposed classification of mandibular impacted third molar: A retrospective study. Natl. J. Maxillofac. Surg..

[36] Ishak M.H., Zhun O.C., Shaari R., Rahman S.A., Hasan M.N., Alam M.K. (2014). Panoramic radiography in evaluating the relationship of mandibular canal and impacted third molars in comparison with cone-beam computed tomography. Mymensingh Med. J..

[37] Deshpande P., Guledgud M.V., Patil K. (2013). Proximity of impacted mandibular third molars to the inferior alveolar canal and its radiographic predictors: A panoramic radiographic study. J. Maxillofac. Oral Surg..

[38] Kuzekanani M., Haghani J., Nosrati H. (2012). Root and canal morphology of mandibular third molars in an Iranian population. J. Dent. Res. Dent. Clin. Dent. Prospect..

[39] Park J.B., Kim N., Park S., Ko Y. (2013). Evaluation of number of roots and root anatomy of permanent mandibular third molars in a Korean population, using cone-beam computed tomography. Eur. J. Dent..

[40] Al-Qudah A.A., Bani Younis H.A.B., Awawdeh L.A., Daud A. (2023). Root and canal morphology of third molar teeth. Sci. Rep..

[41] Bokindo I.K., Butt F., Macigo F. (2017). Variant root morphology of third mandibular molar in normal and impacted teeth. Anat. J. Afr..

[42] Pursafar F., Salemi F., Dalband M., Khamverdi Z. (2011). Prevalence of Impacted Teeth and Their Radiographic Signs in Panoramic Radiographs of Patients Referred to Hamadan Dental School in 2009. Avicenna J. Dent. Res..

[43] Tassoker M., Akyüz M. (2022). Frequency of Pericoronal Radiolucency in Impacted Teeth: A Panoramic Radiography Study. EADS.

[44] Sejfija Z., Koçani F., Macan D. (2019). Prevalence of Pathologies Associated with Impacted Third Molars in Kosovar Population: An Orthopanthomography Study. Acta Stomatol. Croat..

[45] Toedtling V., Devlin H., Tickle M., O’Malley L. (2019). Prevalence of distal surface caries in the second molar among referrals for assessment of third molars: A systematic review and meta-analysis. Br. J. Oral Maxillofac. Surg..

[46] Silva H.O., Pinto A.S.B., de Siqueira Rego M.R., Gois J.F., de Araujo T.L.C., Mendes J.D.P. (2015). Dental caries on distal surface of mandibular second molars. Braz. Dent. Sci..

[47] Oyebunmi B.R. (2019). Complications associated with the occurrence of impacted mandibular third molars in Saudi Arabian sub-population: Najran province experience. Am. J. Biomed. Sci. Res..

[48] Pentapati K.C., Gadicherla S., Smriti K., Vineetha R. (2019). Association of impacted mandibular third molar with caries on distal surface of second molar. Pesqui. Bras. Odontopediatr. Clin. Integr..

[49] Syed K.B., Alshahrani F.S., Alabsi W.S., Alqahtani Z.A., Hameed M.S., Mustafa A.B., Alam T. (2017). Prevalence of Distal Caries in Mandibular Second Molar Due to Impacted Third Molar. J. Clin. Diagn. Res..

[50] Marques J., Montserrat-Bosch M., Figueiredo R., Vilchez-Pérez M.A., Valmaseda-Castellón E., Gay-Escoda C. (2017). Impacted lower third molars and distal caries in the mandibular second molar. Is prophylactic removal of lower third molars justified?. J. Clin. Exp. Dent..

[51] Kunwar D., Koirala U., Manandhar A., Subedi S., Gurung N. (2021). Association of Prevalence of Dental Caries in Mandibular Second Molar with Impacted Third Molar. J. Nepal. Health Res. Counc..

